# Estimated Glomerular Filtration Rate Is Associated With an Increased Risk of Death in Heart Failure Patients With Preserved Ejection Fraction

**DOI:** 10.3389/fcvm.2021.643358

**Published:** 2021-04-26

**Authors:** Zhuo Chen, Qian Lin, Jingen Li, Xinyi Wang, Jianqing Ju, Hao Xu, Dazhuo Shi

**Affiliations:** ^1^Cardiovascular Diseases Center, National Clinical Research Center for Chinese Medicine Cardiology, Xiyuan Hospital, China Academy of Chinese Medical Sciences, Beijing, China; ^2^Graduate School, China Academy of Chinese Medical Sciences, Beijing, China; ^3^Department of Cardiology, Dongzhimen Hospital, The First Affiliated Hospital of Beijing University of Chinese Medicine, Beijing, China; ^4^Graduate School, Beijing University of Chinese Medicine, Beijing, China

**Keywords:** heart failure, preserved ejection fraction, estimated glomerular filtration rate, outcome, risk of death

## Abstract

**Background:** Renal dysfunction is associated with adverse cardiovascular outcomes in patients with heart failure (HF), but its impact on patients with heart failure with preserved ejection fraction (HFpEF) remains unclear.

**Methods:** 3,392 subjects of the TOPCAT (Treatment of Preserved Cardiac Function Heart Failure with an Aldosterone Antagonist) trial were assigned to two groups by estimated glomerular filtration rate (eGFR) ≥ 60 ml/min/1.73 m^2^ or 30–59 ml/min/1.73 m^2^. The outcomes, including all-cause death, cardiovascular death and HF hospitalization, were examined by multivariable cox models.

**Results:** Over a median follow-up of 3.4 ± 1.7 years, a total of 524 all-cause deaths, 334 cardiovascular deaths and 440 HF hospitalizations occurred. Compared with patients with eGFR ≥ 60 ml/min/1.73 m^2^, those with eGFR 30–59 ml/min/1.73 m^2^ were associated with an increased risk of the all-cause death [adjusted hazard ratio (HR), 1.47; 95% confidence interval (CI), 1.24–1.76; *P* < 0.001], cardiovascular death (adjusted HR, 1.53; 95% CI: 1.23–1.91; *p* < 0.001), and HF hospitalization (adjusted HR: 1.21; 95% CI: 1.00–1.47; *p* = 0.049) after multivariable adjustment for potential confounders.

**Conclusions:** eGFR 30–59 ml/min/1.73 m^2^ was related to an increased risk of all-cause death, cardiovascular death and HF hospitalization in HFpEF patients.

## Introduction

Heart failure with preserved ejection fraction (HFpEF) accounts for more than 50% of hospitalized patients with heart failure (HF). The mortality and costs of healthcare in HFpEF patients are similar to those in heart failure with reduced ejection fraction (HFrEF) patients (1). Although the mechanisms of pathogenesis of HFpEF have not been clarified, a novel theory from the pathophysiological perspective for HFpEF draws public attention to comorbidity-induced endothelial inflammation, oxidative stress, cardiac hypertrophy and myocardial fibrosis (2, 3). HFpEF is clinically manifested as a complex syndrome caused by multiple comorbidities and inflammatory mediators (4). Chronic kidney disease (CKD), one of the common HFpEF comorbidities, appears to have a great effect on the pathogenesis of HFpEF (5). It is shown that over 50% of HF patients have renal impairment, among them nearly one third had moderate or severe impairment (6). Impaired renal function is related to poor prognosis in patients with HF (7, 8). However, previous studies on the relationship between renal impairment and HF mainly focused on HFrEF. Only a few studies focused on HFpEF with either small sample size (5) or uncertain conclusion (9). Therefore, we analyzed data of TOPCAT (Treatment of Preserved Cardiac Function Heart Failure with an Aldosterone Antagonist) to determine the prognostic importance of renal function in HFpEF patients.

## Methods

### Study Design and Population

We assessed the association between renal function and mortality and hospitalization for HF in patients with HFpEF using data from the TOPCAT trial (10). The design, protocol, and characteristics of the TOPCAT study have been published previously (11, 12). TOPCAT is an international, multicenter, randomized, double-blind, placebo-controlled trial. A total of 3,445 HFpEF patients from 6 countries were included from August 10, 2006 to January 31, 2012, in order to test the efficacy of spironolactone. Eligible patients included those with symptomatic HF and left ventricular (LV) ejection fraction (LVEF) documented ≥45% who had either a hospitalization for HF in the past 12 months or elevated brain natriuretic peptide (BNP; BNP ≥ 100 pg/mL or N-terminal pro-BNP ≥ 360 pg/mL) within 60 days before randomization. Patients were excluded if they had any of the following situations: a severe systemic illness with a life expectancy judged to be <3 years; known infiltrative or hypertrophic obstructive cardiomyopathy or known pericardial constriction; severe pulmonary disease, such as chronic pulmonary disease requiring home oxygen; severe renal dysfunction [defined as an estimated glomerular filtration rate (eGFR) < 30 mL/min/1.73 m^2^ or serum creatinine level ≥ 2.5 mg/dL]; heart transplant; or known chronic hepatic disease (defined as aspartate aminotransferase and alanine aminotransferase levels > 3.0 times the upper limit of the normal range as determined at the local laboratory) (12). Eligible patients were randomly assigned to spironolactone or placebo group at a 1:1 ratio. In the present study, 1.5% patients with missing information regarding eGFR were excluded, which resulted in a final sample of 3,392. The present study was approved by Medical Ethics Committee of Xiyuan Hospital, China Academy of Chinese Medical Sciences (2019XLA043-1). The National Heart, Lung, and Blood Institute (NHLBI) approved our use of the TOPCAT data.

### Renal Function

Renal function was assessed by eGFR. Patients' eGFR were available during the baseline visit. In accordance with guidelines (13), participants were classified into five groups based on the National Kidney Foundation Kidney Disease Outcomes Quality Initiative (NKF KDOQI) 2000 guidelines. Stage 0/1 CKD was defined as eGFR > 90 mL/min/1.73 m^2^; stage 2 CKD as eGFR 60–89 mL/min/1.73 m^2^; stage 3 CKD as eGFR 30–59 mL/min/1.73 m^2^; stage 4 CKD as eGFR 15–29 mL/min/1.73 m^2^; and stage 5 CKD as eGFR < 15 mL/min/1.73 m^2^ or if the participant was on dialysis. To determine a cut-off, a restricted cubic spline model was also used (shown in Supplementary Figure 1). Moderate renal impairment in this analysis was defined as eGFR 30–59 mL/min/1.73 m^2^ (which corresponds to stage 3 CKD). Normal or slightly impaired renal function refer to baseline eGFR ≥ 60 ml/min/1.73 m^2^ (which corresponds to normal renal function or stage 1/2 CKD).

### Outcome

The outcomes included all-cause death, cardiovascular (CV) death and hospitalization for HF. All-cause Death included the composite of CV and non-CV death. CV death was defined as death caused by myocardial infarction, stroke, sudden death, pump failure, pulmonary embolism, or cardiovascular procedure-related events. Non-CV death was defined as death from non-cardiovascular events, including infection and malignancy. Hospitalization for HF was defined as unexpected presentation to an acute care facility requiring an overnight hospitalization with exacerbation of HF. All Outcomes were adjudicated according to pre-specified criteria by a clinical endpoint committee of TOPCAT (11).

### Potential Confounders

Potential confounders at baseline were: basic information (age, sex, race); lifestyle (smoking status, alcohol intake); history of diseases [hypertension, atrial fibrillation, myocardial infarction (MI), coronary artery bypass graft surgery, percutaneous coronary intervention, implanted cardioverter-defibrillator (ICD), chronic obstructive pulmonary disease (COPD), peripheral vascular disease (PAD), implanted pacemaker, dyslipidemia, diabetes mellitus (DM), previous hospitalization for HF, stroke]; New York Heart Association (NYHA) functional class; physical examination [body mass index (BMI), heart rate (HR), systolic blood pressure (SBP), diastolic blood pressure (DBP)], laboratory data [ejection fraction (EF), leukocyte count, hematocrit (HCT), hemoglobin (HB), platelet count (PLT)]; medications [aspirin, beta-blockers, angiotensin-converting enzyme inhibitors (ACEI) or angiotensin II receptor blockers (ARB), calcium-channel blockers (CCB), diuretics, long-acting nitrate, lipid-lowering drugs]; randomization arm (spironolactone or placebo); Race was classified as white, black, or others. Smoking status was classified as never smoked, former smoker, or current smoker. Alcohol intake was categorized as 0, 1–5, 6–10, or ≥11 drinks per week.

### Statistical Analysis

Demographic data between participants with eGFR ≥ 60 ml/min/1.73 m^2^ and those with eGFR 30–59 ml/min/1.73 m^2^ were compared. Descriptive statistics were presented as numbers, proportions (%), or mean ± SD or median (interquartile range). Continuous variables were compared using Student's *t*-test, categorical variables were compared using chi-square (χ^2^) tests. And Mann-Whitney U-test was used for non-parametric test of two independent samples. We examined the effect of eGFR on the risk of each outcome (all-cause death, CV death and hospitalization for HF) using Kaplan-Meier survival curves and tested for significance using the log rank test. The assessment of the proportional hazard hypothesis in survival was presented by the scaled Schoenfeld residuals. Cox proportional hazard models were used to examine the risk of each outcome associated with different eGFR categories. We analyzed and compared hazard ratios (HRs) for each outcome with 95% confidence intervals (CIs) in the two groups.

Multivariable models were performed to explore the association between eGFR and each outcome, respectively. In model 1, we adjusted for basic demographics: age, sex, race. In model 2, we included all-cause death and 38 characteristics [age, sex, race, hypertension, atrial fibrillation, MI, stroke, CABG, PCI, COPD, previous hospitalization for HF, PAD, ICD, implanted pacemaker, dyslipidemia, DM, NYHA functional class, smoking status, alcohol intake, BMI, EF, HR, SBP, DBP, WBC, HCT, HB, PLT, ACEI/ARB, beta blockers, CCB, diuretics, aspirin, nitrate, lipid-lowering drug, randomization arm (spironolactone or placebo) and eGFR groups] in baseline to build the least absolute shrinkage and selection operator (LASSO) which is suitable for the regression of high-dimensional data (shown in Supplementary Figure 2 for details). And finally the following parameters were adjusted in model 2: age, sex, race, MI, previous hospitalization for HF, smoking status, alcohol intake, EF, HR, diuretics. In model 3, we further included the parameters of model 2 along with stroke, DM, HB, hypertension, atrial fibrillation, ICD, COPD, NYHA class, implanted pacemaker, dyslipidemia, beta blockers, ACE-I/ARB, CCB and randomization arm (spironolactone or placebo).

Subgroup analysis was conducted by cox proportional hazard models to assess the association between eGFR and all-cause mortality in the different subgroups. Two sensitivity analyses were conducted; one with data from Russia and Georgia deleted, and the other with data of patients with mid-range ejection fraction (EF: 45–49%) deleted. And we also conducted competitive risk regression model for cardiovascular death in case of bias caused by non-cardiovascular death (14). Attributive risk calculation was used to elucidate whether various values of eGFR predicted CV and non-CV risk for subpopulation (shown in Supplementary Table 1 for details). Statistical significance, including interaction terms, was defined as *P* < 0.05. Statistical analysis was performed using R version 3.6.1 (the R Foundation for Statistical Computing, Vienna, Austria) (flow chart is shown in Supplementary Materials).

## Results

**Figure 1:**
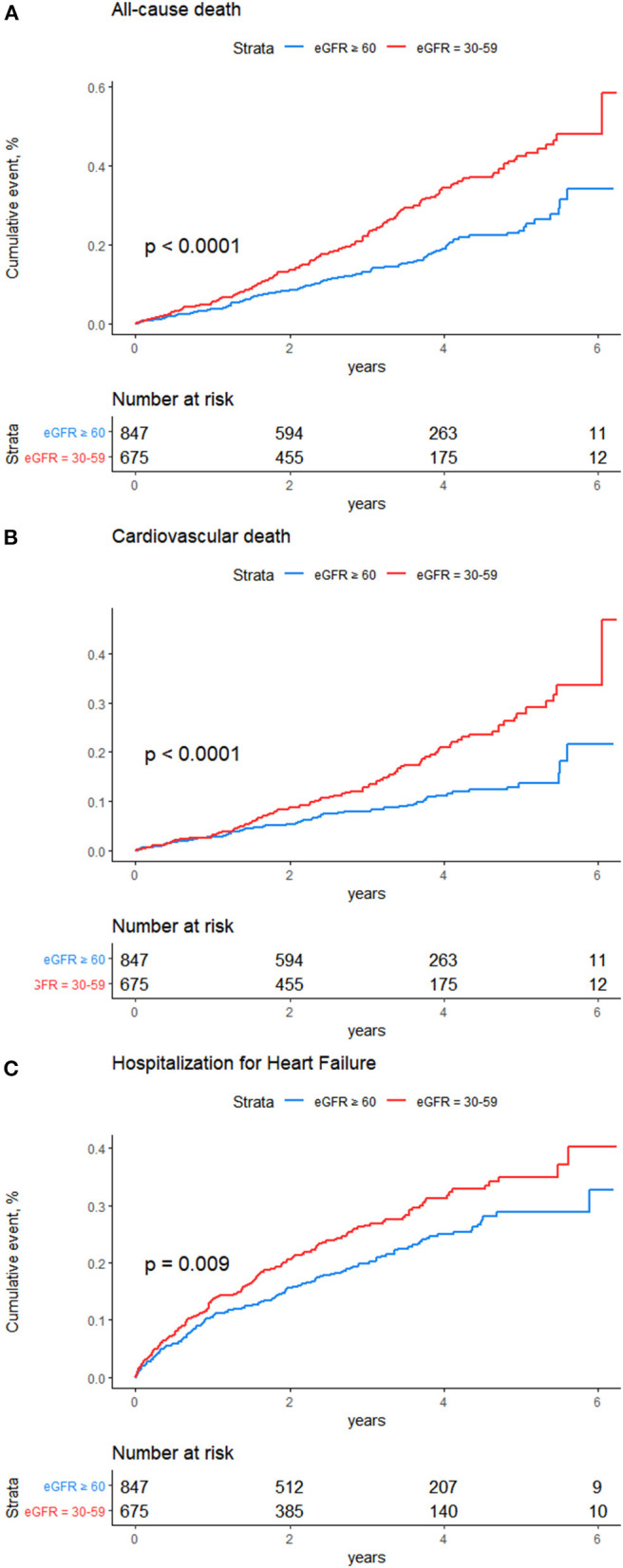
Kaplan-Meier survival curves for events. Rates of **(A)** all-cause mortality, **(B)** cardiovascular death, **(C)** Hospitalization for heart failure.

This analysis included 3,392 patients, among them, 2,080 patients with eGFR ≥ 60 ml/min/1.73 m^2^, 1,312 patients with eGFR 30–59 ml/min/1.73 m^2^. During the follow-up 3.4 ± 1.7 years, there were 524 all-cause death, 334 CV deaths, 440 hospitalization for HF. Kaplan-Meier survival curves and cumulative event rates for all-cause death, CV death and hospitalization for HF in the two groups are shown in Figure 1. All-cause death rate in patients with eGFR ≥ 60 ml/min/1.73 m^2^ and eGFR 30–59 ml/min/1.73 m^2^ were 37 and 60 events per 1,000 person-years, respectively. Characteristics are shown in Table 1. There were statistical differences between the two groups in the following parameters: age, sex, stroke, DM, DBP, WBC, HCT, HB, PLT.

**Table 1:** Baseline clinical characteristics of HFpEF patients with and without renal impairment.

	**eGFR ≥ 60 (*n* = 2,080)**	**eGFR = 30–59 (*n* = 1,312)**	***P-*value**
Age, median (quartile 1–3), year	68 (60–75)	70 (63–77)	<0.001
Male, *n* (%)	1,052 (50.6)	592 (45.1)	0.002
**Race, *n* (%)**			
White	1,867 (89.8)	1,157 (88.2)	0.098
Black	174 (8.4)	116 (8.8)	
Other	39 (1.9)	39 (3.0)	
**History of diseases, *n* (%)**			
Hypertension	1,906 (91.6)	1,198 (91.3)	0.790
Atrial fibrillation	717 (34.5)	483 (36.8)	0.176
Previous myocardial infarction	551 (26.5)	332 (25.3)	0.468
Coronary artery bypass graft surgery	259 (12.5)	177 (13.5)	0.408
Percutaneous coronary intervention	303 (14.6)	192 (14.6)	0.997
Implanted cardioverter defibrillator	23 (1.1)	21 (1.6)	0.278
Chronic obstructive pulmonary disease	257 (12.4)	138 (10.5)	0.116
Peripheral arterial disease	193 (9.3)	123 (9.4)	0.974
Implanted pacemaker	153 (7.4)	114 (8.7)	0.181
Dyslipidemia	1,248 (60.0)	800 (61.0)	0.596
Diabetes mellitus	647 (31.1)	455 (34.7)	0.033
Stroke	144 (6.9)	119 (9.1)	0.027
Previous hospitalization for CHF	1,491 (71.7)	965 (73.6)	0.252
**NYHA class, *n* (%)**			
I or II	1,387 (66.7)	891 (67.9)	0.481
III or IV	693 (33.3)	421 (32.1)	
**Smoking status, *n* (%)**			
current	236 (11.3)	120 (9.1)	0.078
Never	1,070 (51.4)	713 (54.3)	
Former	774 (37.2)	479 (36.5)	
**Alcohol drinks/week, *n* (%)**			
0	1,636 (78.7)	1,010 (77.0)	0.315
1–5	346 (16.6)	226 (17.2)	
6–10	66 (3.2)	57 (4.3)	
≥11	32 (1.5)	19 (1.4)	
BMI, median (quartile 1–3), kg/m^2^	30.78 (26.97–35.76)	31.02 (27.39–35.49)	0.254
HR, median (quartile 1–3), bpm	68 (61–76)	68 (62–76)	0.764
SBP, median (quartile 1–3), mmHg	130 (120–140)	130 (120–139)	0.371
DBP, median (quartile 1–3), mmHg	80 (70–82)	79 (70–80)	0.002
EF, median (quartile 1–3), %	56 (51–61)	57 (52–61)	0.108
Leukocyte count, median (quartile 1–3), k/uL	6.7 (5.6–8.0)	6.8 (5.6–8.2)	0.013
HB, median (quartile 1–3), g/dL	13.5 (12.5–14.7)	12.8 (11–814.0)	<0.001
HCT, median (quartile 1–3), %	40.9 (37.7–44.0)	39.0 (35.9–42.0)	<0.001
PLT, median (quartile 1–3), k/uL	226 (192–262)	220 (186–264)	0.043
**Medications, *n* (%)**			
ACE-I/ARB	645 (31.0)	422 (32.2)	0.504
Beta blockers	683 (32.8)	443 (33.8)	0.602
Calcium channel blockers	316 (15.2)	203 (15.5)	0.864
Diuretics	701 (33.7)	462 (35.2)	0.386
Aspirin	547 (26.3)	369 (28.1)	0.260
Nitrate	137 (6.6)	82 (6.2)	0.751
Lipid lowering drugs	88 (4.2)	63 (4.8)	0.484
**Randomization arm, *n* (%)**			
Spironolactone	1,048 (50.4)	648 (49.4)	0.597

*eGRF, estimated glomerular filtration rate; NYHA, New York Heart Association; BMI, body mass index; HR, heart rate; SBP, systolic blood pressure; DBP, diastolic blood pressure; EF, ejection fraction; HB, Hemoglobin; HCT, Hematocrit; PLT, platelet count; ACE-I, angiotensin-converting enzyme inhibitors; ARB, angiotensin II receptor blockers*.

**Figure 2:**
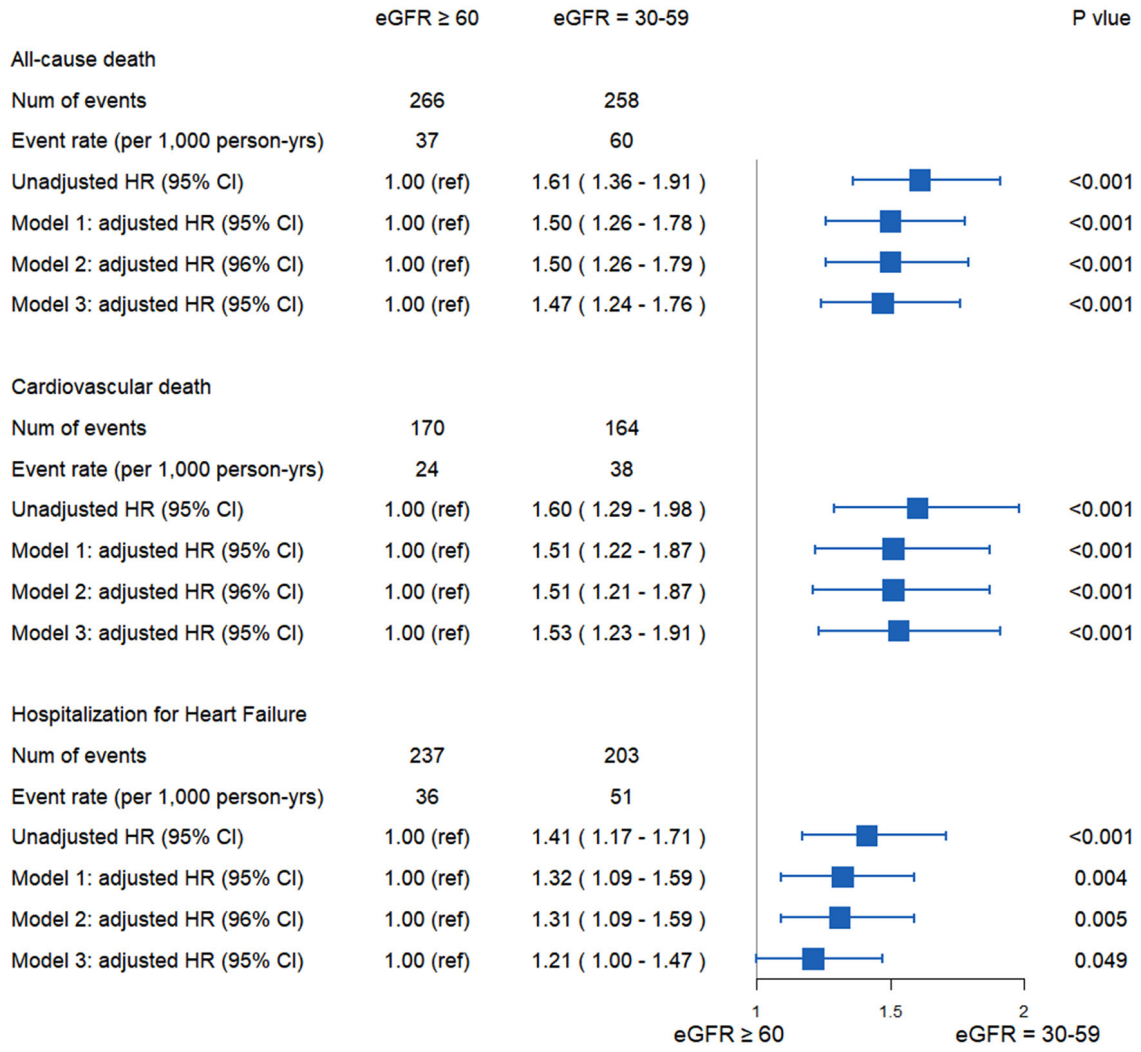
All-cause death, cardiovascular death, and hospitalization for heart failure in HFpEF patients according eGFR. In model 1, the following parameters were adjusted: age, sex, race. In model 2, the following parameters were adjusted: age, sex, race, MI, previous hospitalization for heart failure, smoking status, alcohol intake, EF, HR, diuretics. In model 3, the following parameters were adjusted: the parameters of model 2 along with stroke, DM, HB, hypertension, atrial fibrillation, ICD, COPD, NYHA class, implanted pacemaker, dyslipidemia, beta blockers, ACE-I/ARB, CCB and randomization arm (spironolactone or placebo). CI, confidence interval; HFpEF, heart failure with preserved left ventricular ejection fraction; HR, hazard ratio; MI, myocardial infarction; EF, ejection fraction; HR, heart rate; DM, diabetes mellitus; HB, hemoglobin; ICD, implanted cardioverter-defibrillator; COPD, chronic obstructive pulmonary disease; NYHA, New York Heart Association; ACEI, angiotensin-converting enzyme inhibitors; ARB, angiotensin II receptor blockers; CCB, calcium-channel blockers.

The risk of all-cause death was significantly higher in patients with eGFR 30–59 ml/min/1.73 m^2^ than in those with eGFR ≥ 60 ml/min/1.73 m^2^ [unadjusted HR: 1.61; 95% CI: 1.36–1.91; *p* < 0.001 (Figure 1A); model 1, adjusted HR: 1.50; 95% CI: 1.26–1.78; *p* < 0.001; model 2, adjusted HR: 1.50; 95% CI: 1.26–1.79; *p* < 0.001; and model 3, adjusted HR: 1.47; 95% CI: 1.24–1.76; *p* < 0.001]. The risk of CV death was also higher in group with eGFR 30–59 ml/min/1.73 m^2^ than the other group [unadjusted HR: 1.60; 95% CI: 1.29–1.98; *p* < 0.001 (Figure 1B); model 1, adjusted HR: 1.51; 95% CI: 1.22–1.87; *p* < 0.001; model 2, adjusted HR: 1.51; 95% CI: 1.21–1.87; *p* < 0.001; and model 3, adjusted HR: 1.53; 95% CI: 1.23–1.91; *p* < 0.001]. The risk of hospitalization for HF was higher in group with eGFR 30–59 ml/min/1.73 m^2^ than the other group [unadjusted HR:1.41; 95% CI: 1.17–1.71; *p* < 0.001 (Figure 1C); model 1, adjusted HR: 1.32; 95% CI: 1.09–1.59; *p* = 0.004; model 2, adjusted HR:1.31; 95% CI: 1.09–1.59; *p* = 0.005; and model 3, adjusted HR: 1.21; 95% CI: 1.00–1.47; *p* =0.049] (Figure 2).

**Figure 3:**
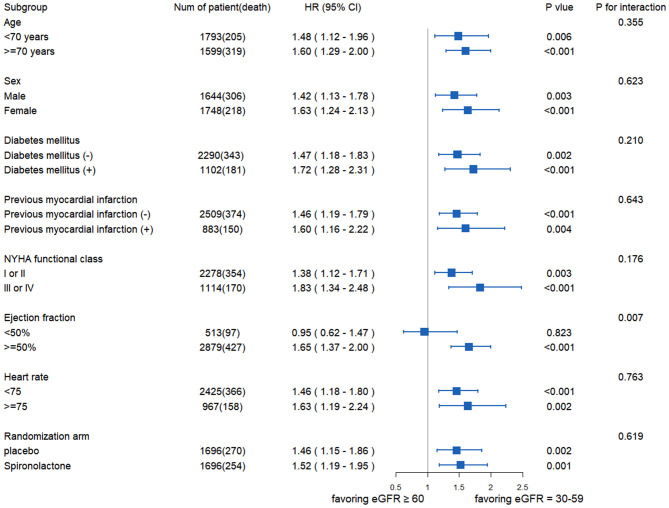
The association between eGFR and all-cause mortality in the different subgroups. NYHA, New York Heart Association.

Figure 3 exhibited the relationship between eGFR and all-cause death in the different subgroups. No statistically significant interaction was found between eGFR and age, sex, diabetes, MI, NYHA functional class, HR, or medical treatment except for EF. There was no statistical difference in the risk of all-cause mortality between the two groups when EF <50% (HR: 0.95; 95% CI: 0.62–1.47; *p* = 0.823).

To further verify our finding, we conducted the sensitivity analysis. In a competing risk analysis, the association of death with cardiovascular reason didn't statistically differ among causes of death (HR: 1.51; 95% CI: 1.21–1.88; *p* < 0.001) (shown in Supplementary Figure 3 for details). We excluded the data related to Russia and Georgia because of the known significant differences in outcomes due to region, and found that the risk of hospitalization for HF was slightly higher in patients with eGFR 30–59 ml/min/1.73 m^2^ than those with eGFR ≥ 60 ml/min/1.73 m^2^, but no statistical significance was found (shown in Supplementary Figure 4 for details). In addition, sensitivity analysis with data which related to HF with middle range ejection fraction(EF: 45–49%) deleted, the same conclusion was drawn that eGFR 30–59 ml/min/1.73 m^2^ was related to higher risk of all-cause death, cardiovascular death and hospitalization for HF in HFpEF patients (shown in Supplementary Figure 5 for details).

## Discussion

This analysis from TOPCAT indicated that the eGFR 30–59 ml/min/1.73 m^2^ was associated with an increased risk of all-cause death, CV death and hospitalization for HF in HFpEF patients.

In the general population, a meta-analysis which provided quantitative data for CKD definition demonstrated that patients with eGFR < 60 mL/min/1.73 m^2^ had an increased risk of mortality (15). For HF patients, the risk of 1 year and in-hospital mortality was shown to be increased in patients with eGFR < 60 ml/min/1.73 m^2^ compared with those with eGFR > 60 ml/min/1.73 m^2^, too (16, 17). Several secondary analyses of clinical trials, such as Coordinating Study Evaluating Outcome of Advising and Counseling in Heart Failure (COACH) (18) or Randomized Aldactone Evaluation Study (RALES) (19), also showed that HF patients had a high incidence of renal function deterioration which resulted in poor prognosis (20). While, was the association between impaired renal function and risk of mortality different in HFrEF and HFpEF? It was demonstrated that impaired renal function was not associated with LVEF (21, 22). Mortality in patients with HFrEF or HFpEF was significantly associated with renal function (23), every 1 ml/min decrease in creatinine clearance raised patients' mortality by 1% (24).

When it comes to HFpEF, a prospective study indicated that most HFpEF patients had low level of eGFR (<60 ml/min/1.73 m^2^) which was associated with a raised risk of 7-year total death (unadjusted HR: 1.43; 95% CI: 1.10–1.86; *p* = 0.007) and cardiovascular death (unadjusted HR: 1.57; 95% CI: 1.13–2.19; *p* = 0.007), after adjusting for the covariates, the result was still statistically different. Unfortunately, the non-negligible drawback is the small size (25). On the contrary, a Swedish registry study divided HF into preserved ejection fraction heart failure [(EF ≥ 50%), HFpEF], middle range ejection fraction heart failure [(EF: 40–49%), HFmrEF], reduced ejection fraction heart failure [(EF < 40%), HFrEF]. Among all the patients, CKD was significantly associated with 1-year mortality in HFrEF and HFmrEF than in HFpEF (HR: 1.49; 95% CI: 1.42–1.56; and HR: 1.51; 95% CI: 1.40–1.63; and HR: 1.32; 95% CI: 1.24–1.42; *P* for interaction <0.001). Although the incidence of CKD in HFpEF was higher than that in HFmrEF and HFrEF, CKD had little influence on mortality and prognosis in HFpEF compared to in HFmrEF and HFrEF (9). This raised a big concern whether the impaired renal function led to poor prognosis in HFpEF.

In this study, previous findings were extended by showing a definite association between moderately impaired renal function and an increased risk of all-cause death, CV death and hospitalization for HF in HFpEF patients. First, it was noteworthy that we defined moderately impaired renal function as 30–59 ml/min/1.73 m^2^, excluding severe renal damage or kidney failure. Our results indicated that moderately impaired renal function was also closely related to an increased risk of all-cause death, cardiovascular death and HF hospitalization in HFpEF patients. Second, it was demonstrated that no significant interaction was found between eGFR and all-cause death in the different subgroups except for EF. Third, our research excluded competing risk of cardiovascular and non-cardiovascular mortality. Fourth, the risk of hospitalization for HF was slightly higher in HFpEF patients with eGFR 30–59 ml/min/1.73 m^2^ than those with eGFR ≥ 60 ml/min/1.73 m^2^; however, statistical significance was lost after excluding data from Russia and Georgia. Therefore, the relation between eGFR 30–59 ml/min/1.73 m^2^ and risk of hospitalization for HF needed to be treated with caution. Fifth, according to the latest definition of HFpEF, data with EF 45–49% were deleted in sensitivity analysis, and it could still be confirmed that eGFR 30–59 ml/min/1.73 m^2^ was related to higher risk of all-cause death, cardiovascular death and hospitalization for HF in HFpEF patients. In general, subgroup and sensitivity analyses strengthened our research regarding baseline eGFR and adverse prognosis in HFpEF.

The mechanisms that link renal function damage and poor prognosis in patients with HFpEF are unclear. The main cause of HFpEF is hypertension, the other risk factors include myocardial ischemia, diabetes, hyperlipidemia, hypertrophic cardiomyopathy (26). Because of the complex pathophysiological mechanisms of HFpEF, the mechanism of its renal dysfunction may be as follows: (1) According to Frank Starling mechanism, increase of end-diastolic volume and pressure, and increase of central venous pressure leading to renal dysfunction (27). (2) Hemodynamic changes resulting in the initiation of neurohumoral regulation mechanism, and then the inflammatory response and oxidative stress response enhanced (28). (3) Hyperactivity of renin-angiotension-aldosterone system (RAAS) and sympathetic nervous system (SNS) (29). The probable mechanism of renal impairment in leading to undesirable outcomes in HFpEF is deleterious cardiorenal interactions. On the one hand, impaired renal function is related to decreased aortic dilatation and tissue velocity, increased arterial or end-systolic elasticity in early diastolic period and increased left ventricular diastolic stiffness. On the other hand, the deterioration of diastolic function and cardiac mechanical abnormality can lead to the decreased cardiac output or the aggravated congestion of renal vein, resulting in the renal function damage in HFpEF (30).

Several limitations should be noted. First, because this study was an observational study, we could not adjust all potential confounding factors to eGFR in our multivariate models. Second, eGFR was only evaluated at baseline, we were unable to illustrate the relation between worsening renal function (reductions in eGFR or increases in creatinine) and adverse outcomes during the follow-up period. In addition, renal function was assessed by eGFR, but the creatinine was measured in local laboratory rather than central laboratory. Third, it would seem important to adjust for baseline proteinuria in model 2, but we excluded the related data from our analyses due to the deficiency of data.

## Conclusions

This study demonstrated that eGFR 30–59 ml/min/1.73 m^2^ was associated with all-cause death, CV death and hospitalization for HF in patients with HFpEF. Further studies are needed to explore the relationship between worsening renal function and adverse outcomes in HFpEF patients.

## Data Availability Statement

Our article is based on a public database. The data we applied for should be deleted within the specified period. Requests to access these datasets should be directed to https://biolincc.nhlbi.nih.gov/.

## Ethics Statement

The studies involving human participants were reviewed and approved by Medical Ethics Committee of Xiyuan Hospital, China Academy of Chinese Medical Sciences (2019XLA043-1). The patients/participants provided their written informed consent to participate in this study.

## Author Contributions

HX and DS designed the research. ZC wrote the manuscript. QL analyzed data. XW applied data from TOPCAT. JL and JJ assisted in analyzing data. All authors contributed to the article and approved the submitted version.

## Conflict of Interest

The authors declare that the research was conducted in the absence of any commercial or financial relationships that could be construed as a potential conflict of interest.
