# Supplementary material for: Estimated Glomerular Filtration Rate Is Associated With an Increased Risk of Death in Heart Failure Patients With Preserved Ejection Fraction
Source: Front Cardiovasc Med. 2021 Apr 26;8:643358. doi: 10.3389/fcvm.2021.643358 (PMC8107393; doi:10.3389/fcvm.2021.643358)
Supplement: Supplementary file 1 [file Data_Sheet_1.docx]

**Supplementary Materials**
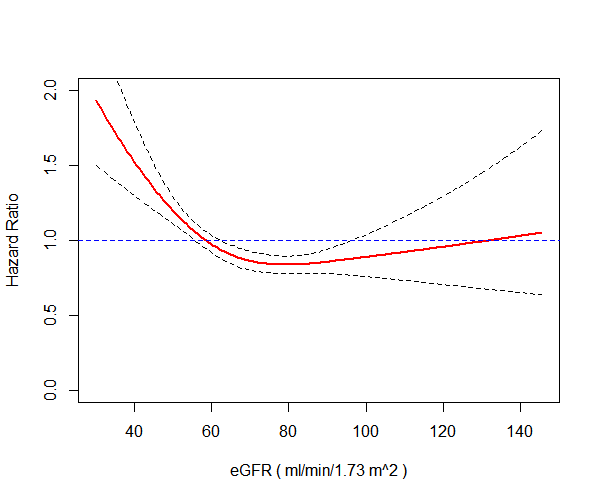


**Figure 1 Risk of All-cause Death for Heart Failure with Preserved Ejection Fraction(HFpEF) across Estimated Glomerular Filtration Rate(eGFR).**

We showed the relationship between the hazard ratio of all-cause death and eGFR using a restricted cubic spline model. As shown in the figure, the optimal cut-point is about 60 ml/min/1.73m^2^. When eGFR less than 60 ml/min/1.73m^2^, the lower eGFR is, the higher the risk of all-cause death for HFpEF is. In addition, according to the clinical stages of chronic kidney disease (CKD), we decided to divide the subjects into two groups: participants with eGFR≥60ml/min/1.73 m^2^ and those with eGFR 30-59 ml/min/1.73 m^2^.


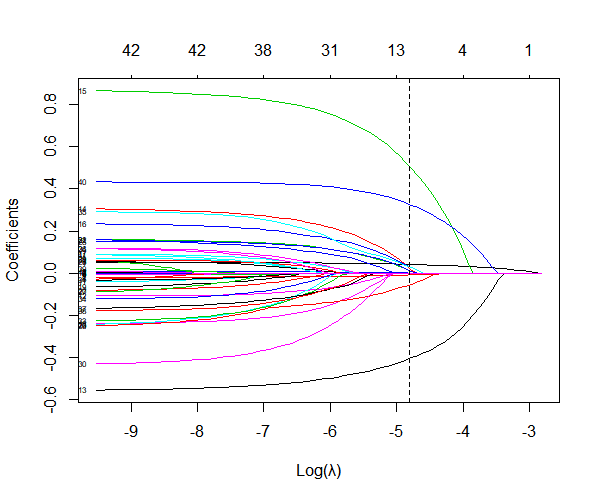


**Figure 2 LASSO Coefficient Profiles Plot of the 38 Baseline Features**

The least absolute shrinkage and selection operator (LASSO) binary logistic regression model was used to screen 38 baseline features. The coefficient of the model changes with the penalty value λ. The position of the dotted vertical lines is the optimal value of λ, which is obtained by displaying the relationship with Area Under Curve (AUC) using 10-fold cross-validation via minimum criteria. When λ value is 0.008, namely log (λ) is - 4.8, AUC area is the largest, and the coefficients of 11 features are not compressed to 0, which are age, sex, race, MI, previous hospitalization for heart failure, smoking status, alcohol intake, EF, HR, diuretics and eGFR.


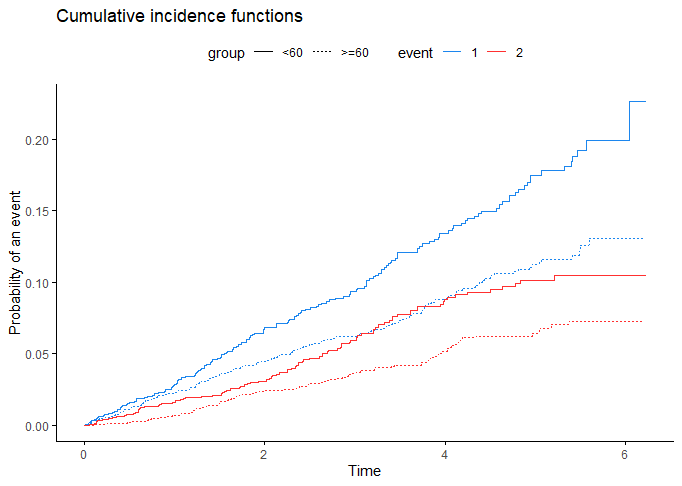


**Figure 3 Cumulative Incidence Curves for Competing Risks for Cardiovascular and Non-cardiovascular Death**

Solid line for eGFR<60, dotted line for eGFR≥60. Event 1 for cardiovascular death，Event 2 for non-cardiovascular death.

The following parameters were adjusted: age, sex, race, MI, previous hospitalization for heart failure, alcohol intake, smoking status, EF, HR, diuretics, stroke, DM, DBP, leukocyte count, HCT, HB, PLT, Hypertension, atrial fibrillation, CABG, PCI, ICD, COPD, PAD, NYHA class, Implanted pacemaker, dyslipidemia, aspirin, beta blockers, ACE-I/ARB, CCB, Lipid-lowering drugs, warfarin and long-acting nitrate.


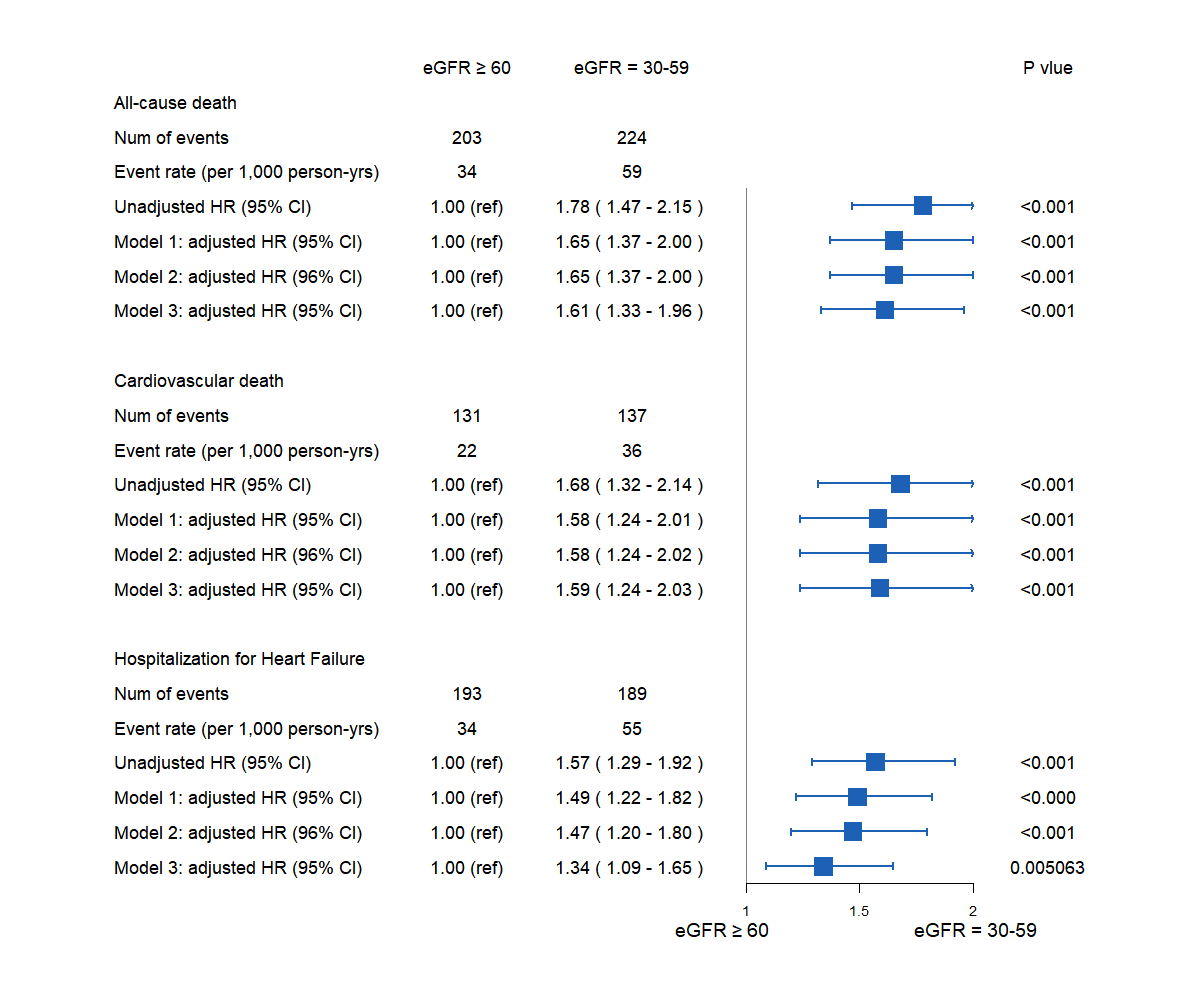


**Figure 4 All-Cause death, Cardiovascular death and Hospitalization for Heart Failure in HFpEF Patients according eGFR (EF ≥ 50%)**

After multivariable adjustment, the risk of all-cause mortality was significantly higher in patients with eGFR 30-59 ml/min/1.73 m^2^ than in those with eGFR≥60 ml/min/1.73 m^2^ (unadjusted HR: 1.78; 95%CI: 1.47 to 2.15; p <0.001; model 1, adjusted HR: 1.65; 95% CI: 1.37 to 2.00; p <0.001; model 2, adjusted HR: 1.65; 95% CI: 1.37 to 2.00; p < 0.001; and model 3, adjusted HR: 1.61; 95% CI: 1.33 to 1.96; p < 0.001). The risk of CV death was also higher in patients with eGFR 30-59 ml/min/1.73 m^2^ than in those with eGFR ≥ 60 ml/min/1.73 m^2^ (unadjusted HR: 1.68; 95%CI: 1.32 to 2.14; p <0.001; model 1, adjusted HR: 1.58; 95% CI: 1.24 to 2.01; p <0.001; model 2, adjusted HR: 1.58; 95% CI: 1.24 to 2.02; p < 0.001; and model 3, adjusted HR: 1.59; 95% CI: 1.24 to 2.03; p < 0.001). The risk of hospitalization for HF was significantly higher in patients with eGFR 30-59 ml/min/1.73 m^2^ than in those with eGFR≥60 ml/min/1.73 m^2^ (unadjusted HR: 1.57; 95% CI: 1.29 to 1.92; p <0.001; model 1, adjusted HR: 1.49; 95% CI: 1.22 to 1.82; p < 0.001; model 2, adjusted HR:1.47; 95%CI: 1.20 to 1.80; p < 0.001; and model 3, adjusted HR: 1.34; 95% CI: 1.09 to 1.65; p =0.005)


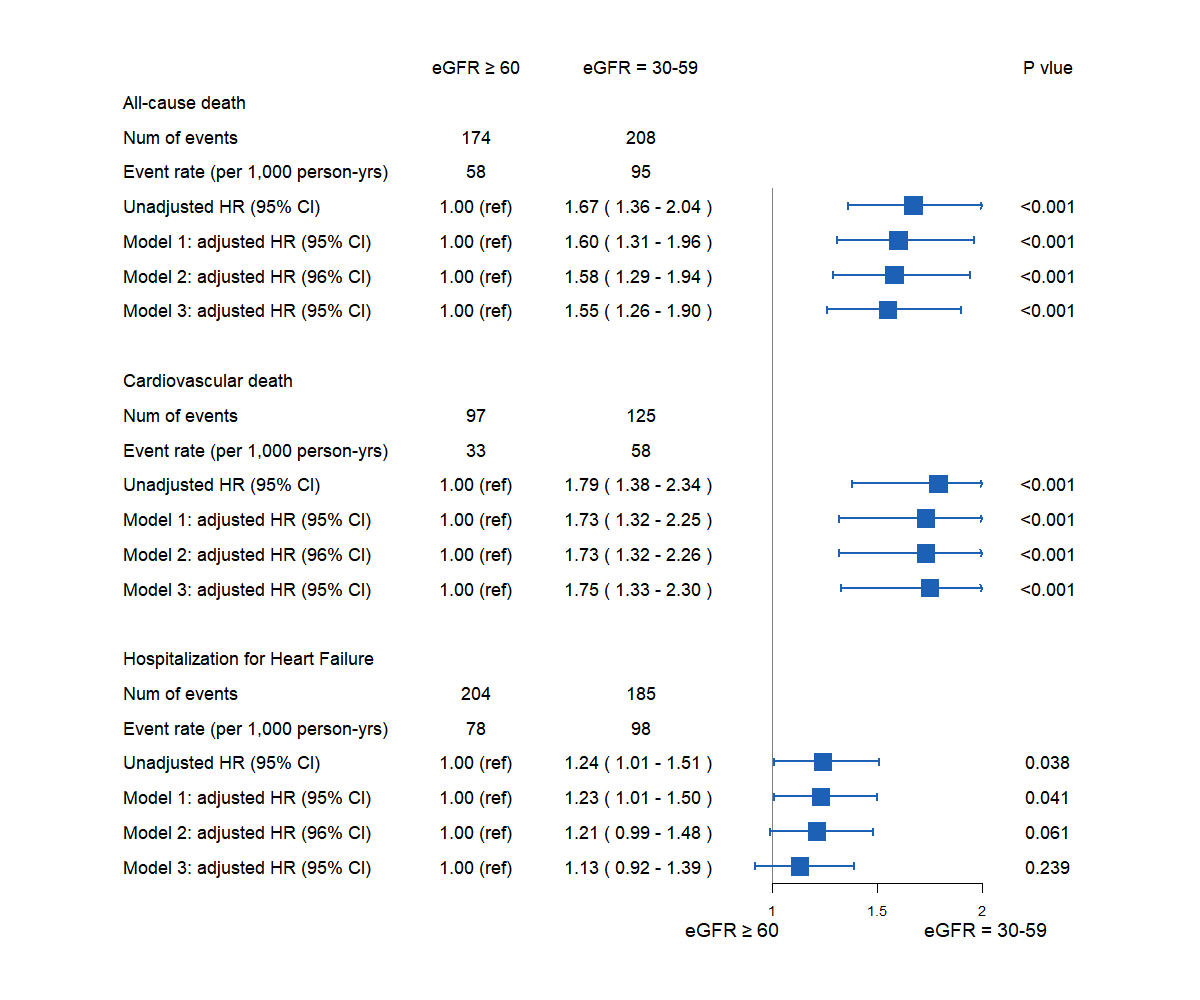


**Figure 5 All-Cause death, Cardiovascular death and Hospitalization for Heart Failure in HFpEF Patients according eGFR (excluding data related to Russia and Georgia)**

After multivariable adjustment, the risk of all-cause mortality was significantly higher in patients with eGFR 30-59 ml/min/1.73 m^2^ than in those with eGFR≥60 ml/min/1.73 m^2^ (unadjusted HR: 1.67; 95% CI: 1.36 to 2.04; p <0.001; model 1, adjusted HR: 1.60; 95% CI: 1.31 to 1.96; p <0.001; model 2, adjusted HR: 1.58; 95% CI: 1.29 to 1.94; p < 0.001; and model 3, adjusted HR: 1.55; 95% CI: 1.26 to 1.90; p < 0.001). The risk of CV death was also higher in patients with eGFR 30-59 ml/min/1.73 m^2^ than in those with eGFR ≥ 60 ml/min/1.73 m^2^ (unadjusted HR: 1.79; 95% CI: 1.38 to 2.34; p < 0.001; model 1, adjusted HR: 1.73; 95% CI: 1.32 to 2.25; p <0.001; model 2, adjusted HR: 1.73; 95% CI: 1.32 to 2.26; p < 0.001; and model 3, adjusted HR: 1.75; 95% CI: 1.33 to 2.30; p < 0.001). The risk of hospitalization for HF was slightly higher in patients with eGFR 30-59 ml/min/1.73 m^2^ than in those with eGFR≥60 ml/min/1.73 m^2^, but no statistical significance was attained. (unadjusted HR: 1.24; 95%CI: 1.01 to 1.51; p = 0.038; model 1, adjusted HR: 1.23; 95% CI: 1.01 to 1.50; p = 0.041; model 2, adjusted HR:1.21; 95% CI: 0.99 to 1.48; p = 0.061; and model 3, adjusted HR: 1.14; 95% CI: 0.92 to 1.39; p =0.239)

**Table 1 Analysis of PAR of eGFR for subpopulation**

| Subgroup | PAR | 95%CI | *p* |
| --- | --- | --- | --- |
| Age |  |  |  |
| <70 years | 15.60% | 4.1% - 25.8% | 0.009 |
| >=70 years | 20.10% | 10.1% - 29% | <0.001 |
|  |  |  |  |
| Sex |  |  |  |
| Male | 13.90% | 4.1% - 22.7% | 0.007 |
| Female | 21.20% | 8.6% - 32% | 0.002 |
|  |  |  |  |
| Diabetes mellitus |  |  |  |
| Diabetes mellitus (-) | 13.80% | 4.5% - 22.2% | 0.004 |
| Diabetes mellitus (+) | 22.30% | 8.2% - 34.2% | 0.003 |
|  |  |  |  |
| Previous myocardial infarction |  |  |  |
| Previous myocardial infarction (-) | 15.50% | 6.4% - 23.8% | 0.001 |
| Previous myocardial infarction (+) | 19.10% | 4.6% - 31.4% | 0.012 |
|  |  |  |  |
| NYHA functional class |  |  |  |
| I or II | 12.70% | 3.3% - 21.3% | 0.010 |
| III or IV | 24.60% | 11.1% - 36.1 | 0.001 |
|  |  |  |  |
| Ejection fraction |  |  |  |
| <50% | -1.20% | -18.2% - 13.3% | 0.877 |
| >=50% | 21.30% | 12.7% - 29% | <0.001 |
|  |  |  |  |
| Heart rate |  |  |  |
| <75 | 15.50% | 6.2% - 23.9% | 0.002 |
| >=75 | 18.90% | 4.9% - 30.9% | 0.010 |
|  |  |  |  |
| Randomization arm |  |  |  |
| placebo | 15.90% | 5.3% - 25.4% | 0.004 |
| Spironolactone | 17.60% | 6.3% - 27.5% | 0.003 |

* PAR: population attributable risk
