# Supplementary figures and images for: Estimated Glomerular Filtration Rate Is Associated With an Increased Risk of Death in Heart Failure Patients With Preserved Ejection Fraction
Source: Front Cardiovasc Med. 2021 Apr 26;8:643358. doi: 10.3389/fcvm.2021.643358 (PMC8107393; doi:10.3389/fcvm.2021.643358)

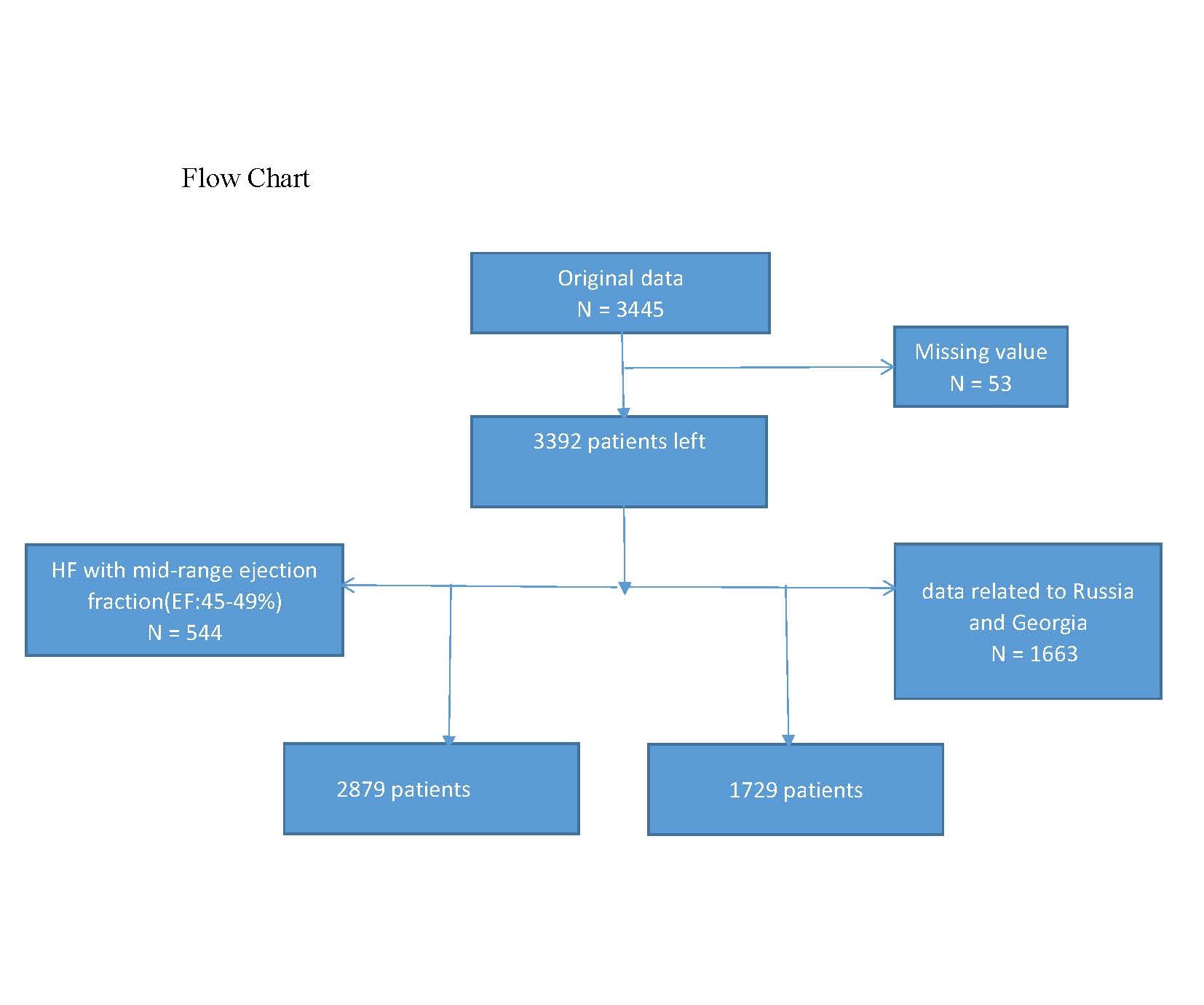

Supplement: Supplementary file 2 [file Image_1.jpg]
